# Circulating tumor cells: A promising marker of predicting tumor response in rectal cancer patients receiving neoadjuvant chemo-radiation therapy

**DOI:** 10.18632/oncotarget.10875

**Published:** 2016-07-28

**Authors:** Wenjie Sun, Guichao Li, Juefeng Wan, Ji Zhu, Weiqi Shen, Zhen Zhang

**Affiliations:** ^1^ Department of Radiation Oncology, Fudan University Shanghai Cancer Center, Shanghai 200032, China; ^2^ Department of Oncology, Shanghai Medical College, Fudan University, Shanghai 200032, China; ^3^ Department of Pathology, Fudan University Shanghai Cancer Center, Shanghai 200032, China

**Keywords:** circulating tumor cells, rectal cancer, neoadjuvant chemo-radiation therapy, prediction

## Abstract

**Purpose:**

The aim of this study was to investigate the role of circulating tumor cells (CTCs) in assessing and predicting tumor response to neoadjuvant chemoradiotherapy (CRT) for patients with locally advanced rectal cancer (LARC).

**Methods:**

A total of 115 patients with T3-4 and/or N+ rectal cancer were enrolled. All patients received neoadjuvant CRT followed by radical surgery after 6-8 weeks. The pathological results after surgery were evaluated according to tumor regression grade (TRG) classification.

**Results:**

Based on TRG score, patients were classified as responders (TRG3-4) and non-responders (TRG0-2). The baseline CTC counts of responders were significantly higher than those of non-responders (44.50±11.94 vs. 37.67±15.45, P=0.012). By contrast, the post-CRT CTC counts of responders were significantly lower than those of non-responders (3.61±2.90 vs. 12.08±7.40, P<0.001). According to ROC analysis, Δ%CTC (percentage difference in CTC counts between baseline and post-CRT) was identified as the stronger predictor to discriminate responders from non-responders (AUC: 0.860). The results of multivariate analysis also indicated that post-CRT CTC counts and Δ%CTC were significantly and independently associated with tumor response to CRT.

**Conclusions:**

The detection of CTCs is a powerful and promising tool for evaluating and predicting responses to neoadjuvant CRT in LARC patients.

## INTRODUCTION

Nowadays neoadjuvant chemoradiation therapy (CRT) combined with radical surgery has become the standard strategy in patients with locally advanced rectal cancer (LARC) [1–5]. Nevertheless, a wide range of treatment responses has been shown after neoadjuvant CRT: some cases obtain complete disappearing of tumor after CRT but others have no response to therapy [6–7]. Previous studies demonstrated that patients with good responses to neoadjuvant CRT would have better prognosis than those without [8–9]. Accordingly, it's very essential to predict accurately the responses to neoadjuvant CRT in order to carry out individualized therapy.

Circulating tumor cells (CTCs), which originate from tumor tissues, are closely associated with tumor invasion and metastasis [10–11]. Thus, detecting and analyzing these tumor cells is very helpful for investigating the intrinsic characteristics of tumors and performing individualized treatment. Nevertheless, the detection and identification of CTCs encounters tremendous difficulties because CTCs are very rare in the blood [12]. In recent years, many techniques with different theories have been performed to detect CTCs [13–17]. Immunomagnetic bead separation based on antibodies for tumor cell surface antigens(e.g. CellSearch system) is one of the most prevalent methods employed to detect CTCs in the clinical settings. The CellSearch system, approved by US FDA, has been widely used in CTC detection for patients with metastatic colorectal, prostate and breast cancer [18]. By use of CellSearch system, the results of many studies demonstrated that the detection of CTCs could be well used to evaluate treatment responses and long-term prognosis for metastatic colorectal cancer patients [19–20]. But for non-metastatic colorectal cancer patients, the positive rate of CTC detection using CellSearch system is too low (about 11%-25%) to further analyze the relationship between CTCs and patients' characteristics and their treatment responses [17, 21]. The main reason for that is probably because this system can not capture a part of CTCs which do not express epithelial antigen [22]. So more efficient and sensitive methods should be used to detect CTCs for non-metastatic patients. Since the sizes of CTCs (15-25μm in diameter) are much larger than those of hematologic cells (7-10μm in diameter) [23], more and more methods based on the differences of cell sizes are used for CTC detection and their capture rates of CTCs are much higher than those using immunomagnetic beads such as CellSearch system [24–25]. In our previous study [26], we introduced our high-performance microfluidic device to detect CTCs. The theory of this device is mainly based on the different sizes between tumor cells and other blood cells. This technique possesses a very high capture rate of CTCs, with fully repeatability. With the high detection rate of our device, we could use it to further analyze and evaluate the potential role of CTCs in clinical settings for non-metastatic cancer patients.

The results of some small series of rectal cancer patients undergoing neoadjuvant CRT indicated that responders to CRT had a significantly higher CTC detection rate compared with non-responders and CRT induced a significant decrease in CTC detection rate for responders [27–28]. Therefore, by use of our microfluidic device, we designed this study to further investigate the role of CTCs in evaluating and predicting treatment responses to neoadjuvant CRT in patients with LARC. To our knowledge, this is the largest series of rectal cancer patients to assess the role of CTCs in predicting the responses to neoadjuvant CRT.

## RESULTS

### Patients' characteristics and histopathologic regression

The clinical characteristics of all 115 patients were shown in Table 1. All patients received comprehensive evaluations at baseline and their clinical stages were evaluated as follows: For clinical T stage, 77(67%) and 38(33%) patients were classified as cT3 and cT4, respectively. For clinical N stage, 15(13%), 57(50%) and 43(37%) patients were classified as cN0, cN1 and cN2, respectively. Of all these 115 patients, 65(57%) had abdominal perineal resection (APR), 45(39%) had anterior resection(AR) and 5(4%) had Hartmann's resection. Pathological stages were evaluated based on the specimens of surgery. For pathological T stage, 27(23%), 8(7%), 28(24%), 48(42%) and 4(3%) patients were classified as pT0, pT1, pT2, pT3 and pT4, respectively. For pathological N stage, 79(69%), 24(21%) and 12(10%) were classified as pN0, pN1 and pN2, respectively. According to the criteria of Dworak, the 115 patients were classified as TRG1 in 14 cases (12%), TRG2 in 37 cases (32%), TRG3 in 39 cases (34%) and TRG4 in 25 cases (22%). No TRG0 subset was observed. 25 of 115 patients (22%) were evaluated as pathological complete response (pCR).

**Table 1:** Patients' characteristics (N=115)

Characteristics	N (%)
Age	
Years, median (Range)	54 (18-91)
Sex	
Male	71 (62%)
Female	44 (38%)
Tumor distance from the anal verge	
≤ 7cm	97 (84%)
> 7cm	18 (16%)
Clinical T stage (baseline)	
T3	77 (67%)
T4	38 (33%)
Clinical N stage (baseline)	
N0	15 (13%)
N+	100 (87%)
Surgical procedure	
APR	65 (57%)
AR	45 (39%)
Hartmann's	5 (4%)
TRG score	
0-2	51 (44%)
3-4	64 (56%)
ypCR	
yes	25 (22%)
no	90 (78%)
Pathological T stage (after surgery)	
T0-2	63 (55%)
T3-4	52 (45%)
Pathological N stage (after surgery)	
N0	79 (69%)
N1-2	36 (31%)

APR: abdominal perineal resection; AR: anterior resection;

TRG: tumor regression grade; pCR: pathological complete response

### CTCs in healthy individuals and rectal cancer patients

CTCs were observed in all 115 rectal cancer patients and we could find at least three positive cells in each sample. By contrast, for 30 healthy donors, we could also find a small quantity of positive cells in 3 blood samples, but the counts of these positive cells were no more than two in each sample.

Rectal cancer patients had significantly higher CTC counts than healthy people (41.67±13.97 vs 0.17±0.59 cells/5mL, P<0.05).

By use of ROC analysis, we found that CTC was a good marker to distinguish cancer patients from healthy people. With a cutoff of 3 cells/5mL, the diagnostic power of CTCs showed 100% sensitivity and 100% specificity.

### CTCs and histologic regression

We compared CTCs and other 5 tumor markers before CRT with those after CRT, and the results were shown in Table 2. CTC counts and the levels of CEA, CA199, CA50 and CA242 before CRT were significantly higher than those after CRT (P<0.001). The levels of CA724 also decreased after the completion of CRT, but no significant difference was observed between pre- and post-CRT.

**Table 2:** CTC and other tumor markers before and after CRT

Markers	Pre-CRT	Post-CRT	P value
CTC (cell/5mL)	41.47±13.97	7.37±6.82	<0.001
CEA (μg/L)	20.11±81.89	11.65±41.00	<0.001
CA199 (U/mL)	40.50±120.90	27.51±77.73	<0.001
CA50 (U/mL)	12.01±46.22	7.76±24.15	<0.001
CA724 (U/mL)	7.17±14.91	5.93±10.32	0.326
CA242 (U/mL)	27.29±40.08	17.63±30.03	<0.001

CTC: circulating tumor cells; Pre-CRT: before chemoradiotherapy;

Post-CRT: after chemoradiotherapy

### Pathologic responders vs. non-responders

**Figure 1:**
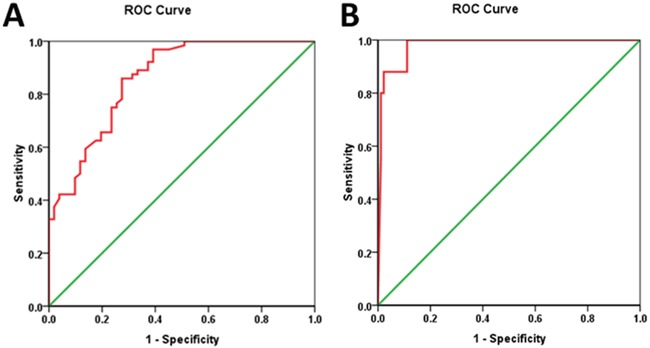
Relationship between CTC counts and tumor regression response **A.** Changes of CTC counts between pre-CRT and post-CRT in TRG 0-2 (N=51) and TRG3-4 (N=64). **B.** Changes of CTC counts between pre-CRT and post-CRT in pCR (N=25) and non-pCR group (N=90) Pre-CRT: before chemoradiotherapy; Post-CRT: after chemoradiotherapy; TRG: tumor regression grade; pCR: pathological complete response.

According to TRG score, 115 patients were regrouped as 64 responders (TRG 3-4) and 51 non-responders (TRG 0-2). As shown in Figure 1A, not only in non-responder group but also in responder group, CTC counts significantly decreased after CRT compared with those before CRT (P<0.001). The responders had significantly higher baseline CTC counts than non-responders (44.50±11.94 vs. 37.67±15.45, P=0.012). By contrast, responders had significantly lower post-CRT CTC counts than non-responders (3.61±2.90 vs. 12.08±7.40, P<0.001). Then we observed and compared Δ%CTC value (percentage difference in CTC counts between pre-CRT and post-CRT) in different TRG score. Significant differences in Δ%CTC (90.62±8.76% in responders vs. 60.03±31.03 in non-responders, P<0.001) were observed between the two groups. For other tumor marker parameters (including Δ%CEA, Δ%199, Δ%50, Δ%724 and Δ%242), we did not find significant difference between these two groups (Table 3).

**Table 3:** Different tumor markers in responders (TRG 3-4) and non-responders (TRG 0-2)

Markers	Non-responders(N=51)	Responders (N=64)	P value
Δ%CTC (%)	60.03±31.03	90.62±8.76	<0.001
Δ%CEA (%)	25.11±34.44	22.32±36.03	0.608
Δ%CA199 (%)	17.11±31.45	11.05±36.73	0.574
Δ%CA50 (%)	12.81±59.77	−1.63±70.52	0.186
Δ%CA724 (%)	−54.39±204.10	−31.03±128.67	0.389
Δ%CA242 (%)	18.30±54.31	14.25±55.14	0.199

Δ%CTC, Δ%CEA, Δ%CA199, Δ%CA50, Δ%CA724, Δ%CA242: percentage difference between pre-CRT and post-CRT for CTC, CEA, CA199, CA50, CA724, CA242, respectively

TRG: tumor regression grade

**Figure 2:**
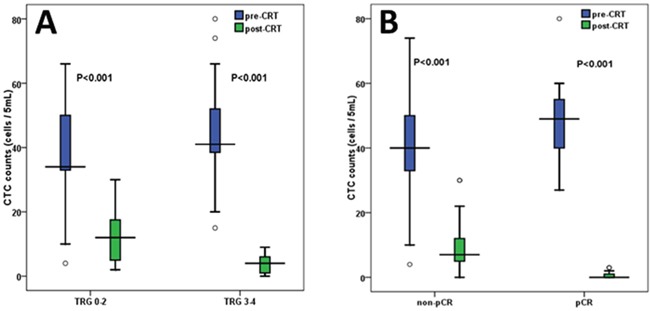
Receiver operating characteristic (ROC) curve analysis for Δ%CTC **A.** Receiver operating characteristic (ROC) curve analysis for Δ%CTC in discriminating responders (TRG 3-4) from non-responders (TRG 0-2). **B.** Receiver operating characteristic (ROC) curve analysis for Δ%CTC in discriminating pCR from non-pCR patients TRG: tumor regression grade; pCR: pathological complete response; Δ%CTC: percentage difference in CTC counts before and after chemoradiotherapy

According to ROC analysis (demonstrated in Figure 2A), Δ%CTC was identified as the stronger predictor to discriminate responders from non-responders compared with other tumor markers (area under the curve, AUC: 0.860). When the cut-off threshold of Δ%CTC was defined as 81.91%, the accuracy of prediction was 78.26%(90/115), with a sensitivity of 85.94%(55/64), a specificity of 68.63%(35/51), a positive predictive value of 77.46%(55/71) and a negative predictive value of 79.55%(35/44).

### Pathologic complete response (pCR) vs. non-pCR

Based on the pathological results after surgery, 115 patients were reclassified as 25 pCR group and 90 non-pCR group. As shown in Figure 1B, not only in pCR group but also in non-pCR group, post-CRT CTC counts were significantly lower than the baseline CTC counts (P<0.001). The patients of pCR group had significantly higher baseline CTC counts than those of non-pCR group (48.04±10.46 vs. 39.64±14.32, P=0.008). On the contrary, the patients of pCR group had significantly lower post-CRT CTC counts than those of non-pCR group (0.72±0.98 vs. 9.21±6.60, P<0.001). Then we observed and analyzed the relationship between Δ%CTC value and pathological results. The patients with pCR had significantly higher Δ%CTC than those without pCR (98.26±2.43% in pCR group vs. 71.16±27.03% in non-pCR group, P<0.001). By contrast, for other tumor marker parameters (including Δ%CEA, Δ%199, Δ%50, Δ%724 and Δ%242), we could not find significant difference between these two groups (Table 4).

**Table 4:** Different tumor markers in pathologic complete response (pCR) group and non-pCR group

Markers	pCR group (N=25)	Non-pCR group (N=90)	P value
Δ%CTC (%)	98.26±2.43	71.16±27.03	<0.001
Δ%CEA (%)	32.61±31.86	21.04±35.84	0.260
Δ%CA199 (%)	13.70±38.11	13.77±33.62	0.972
Δ%CA50 (%)	3.53±45.29	5.12±70.99	0.293
Δ%CA724 (%)	3.42±52.80	−53.84±183.91	0.252
Δ%CA242 (%)	15.57±46.84	16.18±56.77	0.346

Δ%CTC, Δ%CEA, Δ%CA199, Δ%CA50, Δ%CA724, Δ%CA242: percentage difference between pre-CRT and post-CRT for CTC, CEA, CA199, CA50, CA724, CA242, respectively

pCR: pathological complete response

As demonstrated by ROC analysis (Figure 2B), Δ%CTC was identified as a stronger predictor to distinguish patients with pCR and those without [AUC of Δ%CTC: 0.979]. When the cut-off threshold of Δ%CTC was defined as 92.48%, the accuracy of prediction was 90.43%(104/115). The sensitivity, specificity, positive predictive value and negative predictive value were 96%(24/25), 88.89%(80/90), 70.59%(24/34) and 98.77%(80/81), respectively.

### Multivariate analysis

Firstly, logistic regression analysis were performed to find out relevant factors for predicting tumor responses between responders(TRG 3-4) and non-responders(TRG 0-2). At univariate analysis, the covariates with P values <0.2 were as follows: baseline CTC counts(P=0.012), post-CRT CTC counts(P<0.001), Δ%CTC(P<0.001), baseline level of CEA(P=0.173) and CA50(P=0.060), post-CRT level of CEA(P=0.192), CA199(P=0.182) and CA242(P=0.187). Then these covariates were put into the multivariate analysis and the results showed that only post-CRT CTC counts and Δ%CTC were significantly and independently related to treatment response.

Similarly, logistic regression analysis were also carried out to find out relevant factors for predicting tumor responses between pCR and non-pCR patients. At univariate analysis, the covariates with P values <0.2 were as follows: baseline CTC counts(P=0.008), post-CRT CTC counts(P<0.001), Δ%CTC(P<0.001), clinical tumor stage(P=0.130), operation procedure(P=0.129), baseline level of CA50(P=0.028), post-CRT level of CEA(P=0.047) and CA199(P=0.068). Then these covariates were put into the multivariate analysis and the results also indicated that only post-CRT CTC counts and Δ%CTC were significantly and independently related to treatment response.

## DISCUSSION

As mentioned above, there's the need to establish an accurate method to evaluate and predict the effects of neoadjuvant CRT in order to perform the most appropriate treatment for patients with LARC. For patients with pCR or almost pCR, less aggressive therapies such as wait-and-see policy could be performed to spare patients from unnecessary adverse outcomes associated with radical surgery [29–30]. Unfortunately, accurate identification of complete tumor regression remains a significant challenge. The accuracy of conventional methods (e.g. CT/MRI scan, serum CEA) is not reliable enough to assess the treatment responses and guide subsequent treatment strategies [21, 31–32].

In recent years, CTC has been demonstrated as a prognostic marker for colorectal cancer patients [33]. It has been shown that colorectal cancer patients with tumor cells in the blood have a shorter overall survival [34]. In addition to their prognostic value, the detection of CTCs may be useful for assessing and predicting treatment response to CRT. Nevertheless, up till now, only a few studies have investigated the role of CTCs for evaluating responses to neoadjuvant CRT for patients with LARC. Zitt M. et al indicated that responders had an obvious decrease of CTC detection rate after neoadjuvant CRT but there was no noticeable alteration after treatment in non-responders [27]. These results have been confirmed and supported by other studies published in recent years [28, 35]. However, these studies still had their deficiency, including low sample size of patients and less efficient detection techniques of CTCs (e.g., CellSearch system or RT-PCR technique). As we have mentioned above, CellSearch system is not effective enough to well evaluate the role of CTCs in non-metastatic patients. In addition, low detection specificity of RT-PCR technique often makes physicians and biologists doubt the reliability of their results [36]. In our study, we used a high-performance size-based microdevice to detect CTCs. By our device, we further investigated the correlation between CTCs and responses to neoadjuvant CRT in LARC patients. To our knowledge, this is the largest study in this field.

Firstly, in our study, we found that CTC detection showed a high ability to differentiate rectal cancer patients and healthy people. With a cutoff of 3 cells/5mL, the diagnostic power of CTCs showed 100% sensitivity and 100% specificity. Some previous studies also demonstrated that CTC could be used as a good diagnostic marker for various kinds of cancer [37–39]. On the other hand, in our study we also found a few positive cells in some healthy people. Previous studies also indicated similar phenomenon [40–41], but the reason for that is unknown yet.

In our study, the major objective was to assess the role of CTCs in predicting the effects of neoadjuvant CRT in patients with LARC. Firstly, all patients were classified as responders (TRG 3-4) and non-responders (TRG 0-2) based on postoperative pathological results. We found that not only baseline CTC counts but also post-CRT ones had a close association with treatment responses. In addition, Δ%CTC value (percentage difference in CTC counts between pre-CRT and post-CRT) of responders were obviously higher than that of non-responders. According to ROC analysis, Δ%CTC was identified as the stronger predictor to discriminate responders from non-responders, with a higher accuracy of 78.26%(90/115). Meanwhile, we also reclassified patients as pathological complete response (pCR) and non-pCR group to further analyze the relationship between CTCs and treatment response. Similar results were observed: Δ%CTC was also verified as a strong marker to distinguish patients with and without pCR, and the accuracy of prediction was 90.43%. In addition, the results of multivariate analysis also indicated that post-CRT CTC counts and Δ%CTC were significantly and independently associated with tumor response to CRT. Therefore, our results suggest that CTC is a good marker for predicting the effects of neoadjuvant CRT. This might help surgeons to make decision adjustment for subsequent treatment after CRT in individualized therapy. Good responders to neoadjuvant CRT might be performed less aggressive treatment strategies (e.g. wait-and-see policy), which could avoid the toxicities and complications of radical surgery and improve obviously the quality of life of patients. On the other hand, our study also analyzed the association between treatment response and some common circulating tumor markers (including CEA, CA199, CA50, CA724 and CA242). We found the levels of all these tumor markers obviously decreased after CRT. Nevertheless, based on the results of our study, no relationship could be found between treatment response and these tumor markers, which were consistent with the results of previous study [21].

On the other hand, some points should also be addressed for our study. Firstly, our study found a close relationship between CTCs and tumor response to CRT. Δ%CTC obtained an accuracy of prediction of 78.26% for distinguishing different responder groups (TRG 3-4 vs. TRG 0-2), while it also obtained a high accuracy of 90.43% for discriminating pCR from non-pCR group. However, false positives and false negatives could also be observed in our study. False positives can bring about undertreatments of patients who still have viable residual tumor cells while false negatives can cause patients who have obtained complete pathologic responses to receive unnecessary overtreatments. Therefore, at present, CTCs cannot directly be used for predicting treatment response in clinics, and further investigation with larger number of patients should also be performed in the future. Secondly, in our study, we only measured CTC counts at baseline and one week after the completion of neoadjuvant CRT to analyze their association with treatment response. Previous studies also selected similar time points of CTC detection [27–28, 35]. Nevertheless, the optimal time points to detect CTCs haven't been determined till now. The detection of CTCs during the beginning of chemoradiotherapy might be helpful to distinguish responders from non-responders in earlier time and possibly spare non-responders from unnecessary toxicities associated with CRT. So the optimal time points of CTC detection should also be further investigated in the future. Thirdly, in this study, we only evaluated the role of CTCs in predicting short-term effects of neoadjuvant CRT. However, the ultimate goal of chemoradiation therapy is to improve long-term effects including local control rate and even overall survival rate. Therefore, it is also essential to further assess the role of CTCs in predicting long-term effects of CRT. Previous studies indicated that patients with good response to CRT would have better prognosis [8, 9]. So the patients who have an obvious decrease of CTCs after CRT might have better local control rates and survival rates. We will follow up the enrolled patients of our study to perform relevant analysis and update relevant results in the future. Fourthly, pelvic radiotherapy concomitant with 5-Fu/Capecitabine is the typical regimen of neoadjuvant CRT for rectal cancer patients nowadays. In this study, our hospital carried out a clinical trial and oxaliplatin was added to the typical regimen in neoadjuvant CRT. It's hard to say that there's no impact on the results in addition to oxaliplatin compared with typical regimen. Therefore, this point should be considered when comparing with other similar studies about CTCs. Lastly, since these results were based on our newly designed microfluidic device, the reliability of this method should be validated. In the future, we will compare our method with some more standard approaches (e.g. CellSearch and Isoflux) and report relevant information.

## MATERIALS AND METHODS

### Patients' characteristics and blood sample collection

A total of 115 patients, diagnosed with locally advanced rectal cancer, were enrolled in this study between February 2012 and June 2013. The pretreatment evaluation of the patients included a complete clinical history, physical examination, colonoscopy, relevant blood examination, pelvic magnetic resonance imaging and chest/abdominal computed tomography. The recruitment criteria was as follows: pathologically confirmed with rectal adenocarcinoma; diagnosed as locally advanced rectal cancer (cT3-4 and/or N+) by pelvic magnetic resonance imaging; no evidence of distant metastases; tumor distance from anal verge ≤10 cm; no previous chemotherapy, pelvic radiotherapy or surgery for rectal cancer; Karnofsky ≥70 or ECOG 0-2. The characteristics of enrolled patients were shown in Table 1. All patients received neoadjuvant radiotherapy (pelvic radiation therapy with a total dose of 50-55Gy in 25 fractions) and concurrent chemotherapy using Capecitabine (625 mg/m2, twice daily, day1-5/week) and Oxaliplatin (85 mg/m2, day1/week). A standard total mesorectal excision was performed 6-8 weeks after the completion of neoadjuvant CRT. After radical surgery, 5-6 cycles of XELOX(CapeOX) regimen were given to all patients: Oxaliplatin 130 mg/m2 d1, Capecitabine 1000 mg/m2 bid d1-14, q3w. For all these patients, 5ml peripheral blood samples were obtained one week before the initiation of CRT and one week after the completion of CRT, respectively.

As a control group, blood samples were also drawn from 30 healthy volunteers. The definition of healthy volunteers was as follows: no known illness; no fever at the time of draw; no history of malignant disease.

This study was ethically based on the Declaration of Helsinki and the principles of “good clinical practice”. Each patient signed a written informed consent prior to enrollment.

All specimens were drawn into evacuated EDTA-containing blood collection tubes, stored at 4°C and processed within 72h. After centrifugation of peripheral blood at 1500rpm for 10 min, the plasma samples were carefully removed from the upper portion of the supernatant. Red blood cell lysis buffer (0.139 M NH_4_Cl, 0.02 M Tris, pH 7.2) was added to the residual blood specimens and mixed for 45 min at room temperature. Following centrifugation at 1500rpm for 10min, the supernatant was removed and the residual peripheral blood mononuclear cell (PBMC) pellet was resuspended in PBS buffer.

### Device design and fabrication

CTCs were captured by our size-based microfluidic device and the detailed description was shown in our recently published paper [26]. Briefly, the microfluidic device consisted of 80 main channels and 81 side channels, which were arranged in an interdigital manner to minimize the chip footprint. A group of narrow parallel-arranged filter channels were designed to connect each pair of main channel and adjacent side channel. Both main channels and side channels had the cross sections of 50 μm in width and 50 μm in height, while those of filter channels had widths of 20 μm and heights of 5 μm. For each main channel, the right side was connected directly with the sample inlet, and the left side was linked via a filter channel to a waste chamber, with an array of micro-posts that were designed to prevent chamber collapse. For each side channel, the right side was blind and the left one was connected directly with waste chamber. After the blood sample was loaded at the inlet, a negative pressure was applied to the outlet, which aspirated the blood sample into the main channels. Meanwhile, due to pressure difference between the main channel and the side channel, most of the small-sized hematologic cells in whole blood such as erythrocytes and leukocytes could be filtered into their adjacent side channels via filter channels and then eliminated from the waste chamber. The large-sized cells such as tumor cells could not pass through the narrow filter channels and then remained in the main channels. This was the theory of the isolation of CTCs for this device.

Our device was fabricated by bonding a hybrid polydimethylsiloxane (PDMS) slab containing three-dimensional micro-channels with a glass slide. The microfluidic device was fabricated through the well-established multi-layer soft lithography process [42]. Briefly, a two-level master was prepared from a negative photo-resist, SU-8 (Microchem, USA). Subsequently, degassed PDMS (mixed in a 10:1 ratio of PDMS base with curing agent, Sylgard 184, Dow Corning Inc., USA) was cast over the master mold and baked at 90°C for 1h in an oven. After curing, the PDMS slab was carefully peeled off from the master mold. One inlet and one outlet were punched through the PDMS using a needle with a flattened tip. The PDMS slab was then bonded with a glass slide after oxygen plasma treatment, and the process was described as follows: The oxygen plasma bonding was done using dedicated plasma cleaner (PDC-32G-2, Harrick Plasma Corp., USA). The glass slide and PDMS slab were placed in the chamber of plasma cleaner for 50 seconds. After that, PDMS slab and glass slide were taken out of the chamber, and PDMS was placed and pressed on the glass immediately.

### Instrument setup

Before the processing of detection, the concentrations of PBMC samples from patients with rectal cancer were measured by a hemacytometer and diluted in PBS buffer as 5×10^7^ cells/mL. Then, the 0.5-0.6 mL diluted PBMC samples were introduced into the device by pumping. A syringe pump (PHD 22/2000, HAVARD apparatus, Massachusetts, USA) with a 5 mL syringe for waste collecting was connected to the outlet of the device. The inlet of the device was connected to the blood samples via polymer tubing. The syringe pump was turned on and the pressure was adjusted so that the flow rate reached 0.5 mL/h. The blood samples were pumped from the inlet into the microfluidic device for CTC capture and the filtered hematologic constituents such as leukocytes were collected into the waste chamber.

### Identification and enumeration of CTCs by fluorescence microscopy

After the process of CTC capture, tumor cells were identified and distinguished from leukocytes based on morphology and differential antigen expression. Fluorescent reagents were pumped into the device and immunofluorescence reaction was done directly in the channels of the device. Firstly, antibodies to CD45 conjugated to FITC (BD Biosciences, USA) were introduced into the device, followed by incubation for 30 min at 0 °C. Subsequently, a solution of 0.2% Triton X-100 and antibodies to cytokeratin conjugated to phycoerythrin (C-11, Abcam, UK) were pumped into the device and incubated for 10 min and 1 h, respectively. Then, captured cells were mixed with DAPI solution for 20 min. Finally, captured cells were fixed by a solution of 1% paraformaldehyde for 20 min. After these reactions, the device was flushed with 1 mL PBS to remove excess reagents. Captured cells were identified and enumerated using fluorescence microscopy (Olympus America). CTCs were identified according to the stained color and morphological characteristics such as cell size, shape and nuclear size. The cells that stained cytokeratin+/CD45-/DAPI+ and met the phenotypic morphological characteristics were scored as CTCs. An experienced pathologist who was blind to the clinical results comprehensively evaluated each blood sample for CTC identification.

### The detection of serum tumor markers

In this study, we also compared CTCs with some common tumor markers, including serum CEA, CA199, CA50, CA724 and CA242. Serum levels of these tumor markers were assayed using electrochemiluminescence immunoassay technique by cobas e 601 immunoassay analyzers (Roche Diagnostics GmbH). The assays were performed according to the manufacturer's protocols.

### Pathological assessment

For all patients, treatment responses were assessed according to the pathological results after surgery. An experienced pathologist who was blinded to the CTCs and clinical results comprehensively evaluated each surgery specimen. Tumor regression was graded by histological evaluation of the surgical specimens according to the criteria described by Dworak et al [43]. The grade of tumor regression(TRG) was defined as follows:

Grade 0: no regression

Grade 1: dominant tumor mass with obvious fibrosis and/or vasculopathy

Grade 2: dominantly fibrotic changes with few tumor cells or groups (easy to find)

Grade 3: very few tumor cells (difficult to find microscopically) in fibrotic tissue with or without mucous substance

Grade 4: no tumor cells, only fibrotic mass (total regression or response)

### The calculation of CTCs and other tumor markers

CTC counts and the levels of other 5 tumor markers were calculated for further analysis. Δ%CTC, Δ%CEA, Δ%CA199, Δ%CA50, Δ%CA724, Δ%CA242 were defined as percentage difference between pre-CRT and post-CRT for CTC counts and the levels of CEA, CA199, CA50, CA724 and CA242, respectively.

### Statistical analysis

Statistical analyses were performed using SPSS software (version 16.0, SPSS Inc., Chicago, IL). All results were expressed as means ± standard deviations (SD). A Mann-Whitney U test was used in cases of 2 independent samples, whereas the comparisons of related measurements were performed using a Wilcoxon signed rank test. A receiver operating characteristic (ROC) curve was plotted to identify the cut-off value with the highest accuracy for predicting pathologic response. The cut-off value was defined by the point on the ROC curve with the minimum distance from the 0% false positive rate and 100% true positive rate. Sensitivity, specificity and positive and negative predictive values were calculated using standard formulas. All tests were two-sided and were performed at a 5% level of significance. Moreover, to investigate the independent role of CTCs in predicting tumor response, univariate and multivariate logistic regression analysis were performed using the following variables: tumor size, tumor distance from anal verge, tumor morphology, tumor differentiation, clinical tumor stage, tumor markers, operation procedure, baseline and post-CRT CTC counts, Δ%CTC value. Univariate logistic regression analysis was firstly performed to select the variables with P values < 0.2 to be input in the multivariate analysis. Multivariate logistic regression analysis was fit to the data using the forward stepwise mode for variable selection.
